# Growth inhibition of oral mutans streptococci and candida by commercial probiotic lactobacilli - an *in vitro *study

**DOI:** 10.1186/1472-6831-10-18

**Published:** 2010-07-02

**Authors:** Pamela Hasslöf, Maria Hedberg, Svante Twetman, Christina Stecksén-Blicks

**Affiliations:** 1 Paediatric Dentistry, Department of Odontology, Faculty of Medicine, Umeå University, S 901 85 Umeå, Sweden; 2 Oral Microbiology, Department of Odontology, Faculty of Medicine, Umeå University, S 901 85 Umeå, Sweden; 3 Department of Cariology and Endodontics, Faculty of Health Sciences, University of Copenhagen, 3B, Blegdamsvej, DK 2200 Copenhagen, Denmark

## Abstract

**Background:**

Probiotic bacteria are suggested to play a role in the maintenance of oral health. Such health promoting bacteria are added to different commercial probiotic products. The aim of the study was to investigate the ability of a selection of lactobacilli strains, used in commercially available probiotic products, to inhibit growth of oral mutans streptococci and *C. albicans in vitro*.

**Methods:**

Eight probiotic lactobacilli strains were tested for growth inhibition on three reference strains and two clinical isolates of mutans streptococci as well as two reference strains and three clinical isolates of *Candida albicans *with an agar overlay method.

**Results:**

At concentrations ranging from 10^9 ^to 10^5 ^CFU/ml, all lactobacilli strains inhibited the growth of the mutans streptococci completely with the exception of *L. acidophilus *La5 that executed only a slight inhibition of some strains at concentrations corresponding to 10^7 ^and 10^5 ^CFU/ml. At the lowest cell concentration (10^3 ^CFU/ml), only *L. plantarum *299v and *L. plantarum *931 displayed a total growth inhibition while a slight inhibition was seen for all five mutans streptococci strains by *L. rhamnosus *LB21, *L. paracasei *F19, *L. reuteri *PTA 5289 and *L. reuteri *ATCC 55730. All the tested lactobacilli strains reduced candida growth but the effect was generally weaker than for mutans streptococci. The two *L. plantarum *strains and *L. reuteri *ATCC 55730 displayed the strongest inhibition on *Candida albicans*. No significant differences were observed between the reference strains and the clinical isolates.

**Conclusion:**

The selected probiotic strains showed a significant but somewhat varying ability to inhibit growth of oral mutans streptococci and *Candida albicans in vitro*.

## Background

Probiotic bacteria, defined as "live microorganisms which when administered in adequate amounts confer a health benefit on the host" (FAO/WHO 2001), are suggested to play a role in the maintenance of oral health [1,2]. Such health promoting bacteria are added to different commercial dairy products such as milk, cheese and yogurt as well as chewing gums and fruit drinks. Possible actions of probiotic bacteria in the oral environment are competition of binding sites, production of antimicrobial substances and activation and regulation of the immune response [3]. Bacterial antagonism may occur when growth of one bacterial species is hampered by components produced by another species. Lactic acid bacteria produce antimicrobial components [4,5] and some have the ability to produce hydrogen peroxide (H_2_O_2_) that can be toxic to organisms producing little or no H_2_O_2_-scavenging enzymes.

Molecular analyses of the oral microbiota in preschool children have shown that *Streptococcus mutans *is significantly associated with early childhood caries [6]. *Candida albicans *is a persistent member of the oral microbiota in children with caries [7] with a substantial growth response to sucrose exposure [8]. *C. albicans *produce organic acids like pyruvate and acetate and are considered to have a significant contribution to caries pathogenisis [9]. Lactobacilli play a significant role in the oral ecosystem and can be linked with oral disease as well as oral health [10]. Since the discovery by Polonskaya [11] that *L. acidophilus *inhibits growth of certain streptococci *in vitro*, clinical studies have confirmed that probiotic lactobacilli can reduce the counts of salivary mutans streptococci after ingestion of *L. rhamnosus *GG [12,13] and *L. reuteri *[14-16]. Furthermore, naturally occurring *Lactobacillus *species, including *L. paracasei, L. plantarum *and *L. rhamnosus*, may inhibit growth of laboratory strains of mutans streptococci as well as subject's autologous mutans streptococci *in vitro *[17]. Hatakka et al [18] found that a cheese containing a mixture of probiotic bacteria decreased the salivary count of *C. albicans *in a randomized controlled trial among elderly.

The aim of the present study was to investigate the ability of a selection of lactobacilli strains, used in commercially available probiotic products, to inhibit growth of mutans streptococci and *C. albicans in vitro*. The null hypothesis tested was that none of the lactobacilli strains would differ significantly from the other.

## Methods

### Lactobacilli strains and cultivation

Eight strains of probiotic lactobacilli (*L. plantarum *299v, *L. plantarum *931, *L. rhamnosus *GG ATCC 53103, *L. rhamnosus *LB21, *L. paracasei *F19 and *L. reuteri *PTA 5289, *L. reuteri *ATCC 55730 and *L. acidophilus *La5) used in different probiotic products were selected (Table 1). The bacteria were provided by the different producers in pure forms (frozen suspensions or lyophilized) except for *L. acidophilus *La5 that was isolated from A-fil^® ^(Arla Ltd, Stockholm, Sweden). The strains were characterized by the API 50 CH system (BioMérieux^® ^SA, Marcy-l 'Etoile, France) to confirm their identity. The bacteria were initially cultured for 16-20 h on MRS agar (de Man, Rogosa, Sharpe, Oxid, Hampshire, England). A distinct colony of each bacterium was then transferred to 4.5 ml MRS broth for further 16-20 h of incubation.

**Table 1:** Probiotic lactobacilli strains, producer and products used in the present experiments.

Strain	Producer	Product
*L. plantarum *299v	Probi AB, Lund, Sweden	Fruit drink
*L. plantarum *931	Essum, Umeå, Sweden	Fruit drink
*L. rhamnosus *GG ATCC 53103	Valio Ltd, Helsinki, Finland	Yogurt
*L. rhamnosus *LB21	Essum, Umeå, Sweden	Yogurt
*L. paracasei *F19	Arla Ltd, Stockholm, Sweden	Yogurt, Porridge, Gruel
*L. reuteri *PTA 5289	BioGaia, Stockholm, Sweden	Chewing gum, Tablets
*L. reuteri *ATCC 55730	BioGaia, Stockholm, Sweden	Chewing gum, Gruel, Drops, Tablets
*L. acidophilus *La5	Arla Ltd, Stockholm, Sweden	Fermented milk

### Mutans streptococci, candida strains and cultivation

Five strains of mutans streptococci (MS) including both laboratory reference strains (*S. mutans *NCTC 10449, *S. mutans *Ingbritt, and *S. sobrinus *OMZ176) and clinical isolates (*S. mutans *P1:27 and *S. mutans *P2:29) were cultured on blood agar plates (Columbia Blood Agar Base, Alpha BioScience, Baltimore, USA) supplemented with 5% horse blood during 16-20 hours and on the following day, pure colonies of each bacterium were transferred to 2 ml Todd-Hewitt broth (Oxid, Hampshire, England) and incubated for another 16-20 hours. Five strains of *Candida albicans *including both reference strains (*C. albicans *ATCC 28366, *C. albicans *ATCC 10231) and clinical isolates (*C. albicans *1957, *C. albicans *3339 and *C. albicans *GDM8) were cultured on Difco™ Sabourad Maltose Agar (Becton, Dickinson and Complany, Sparks, USA) over night and the following day, pure colonies of each yeast were transferred to 2 ml Difco™ Sabourad Maltose broth and incubated for 16-20 hours. The *C. albicans *strains were characterized by the API Candida (BioMérieux^® ^SA, Marcy-l'Etoile, France). Culturing of lactobacilli and mutans streptococci were performed in anaerobic atmosphere (10% H2, 5% CO2 and 85% N2) in an anaerobe chamber at 37°C, while culturing of *C. albicans *was performed under aerobic conditions at 37°C.

### Agar overlay interference tests

The broth cultures of lactobacilli were serial diluted in tenfold steps. The optical density (OD) was measured at 630 nm using a spectrophotometer at dilution 10^-1 ^(Ultrospec 100 pro, Visible Spectrophotometer, Biochrom Ltd. Cambridge, England). Undiluted suspension and cell suspensions corresponding to approximately 10^9^, 10^7^, 10^5^, and 10^3 ^CFU/ml were used in the inhibition experiments. One ml of each lactobacilli suspension was added into 24 ml molten sterile MRS agar (ca 45°C) and plates were casted. When the agar was set, the plates were incubated at 37°C over night in anaerobic atmosphere. The next day, a second agar layer of 23 ml molted M17 agar supplemented with 10% sterile filtered lactose (May and Baker, Dagenham, England) was casted on top of the MRS agar with grown lactobacilli. The plates were allowed to dry for 3 hours at room temperature. Broth cultures of MS grown for 16-20 hours in Todd-Hewitt broth were diluted in the same medium and the OD was measured at 500 nm and adjusted to 0.2. The suspensions of MS were stamped on the plates with Steer's replicator (CMI-Promex ICN, Pedricktown, USA). The plates were left at room temperature for 1 hour to dry and were subsequently incubated over night at 37°C in the anaerobe chamber. To test the susceptibility of *C. albicans*, 24 ml Difco™ Sabourad Maltose Agar (SAB) was casted on top of the MRS agar with grown lactobacilli. Broth cultures of *C. albicans *grown for 16-20 h in Difco™ Sabourad Maltose Broth were diluted in the same medium and the OD was measured at 500 nm and adjusted to 0.2. The suspensions were then stamped on the plates in the same way as MS (see above). The plates inoculated with *C. albicans *were incubated in air at 37°C. As controls, the MS and *C. albicans *strains were stamped onto agar plates without lactobacilli within the first agar layer.

All assays were made in duplicate and each experiment was repeated on three different occasions. The results of the agar overlay tests were categorized as follows according to Simark-Mattsson et al. [17]: Score 0 = complete inhibition (no visible colonies), Score 1 = slight inhibition (at least one visible colony but definitely smaller amounts then in the control plate), and Score 2 = no inhibition (the same growth as on the control plate). The evaluation of the plates was performed by two independent observers, in case of disagreement a consensus was reached after discussion.

### pH-measurements

As an estimation of the lactobacilli acid production, the surface pH of the MRS plates with M17 and SAB agar was determined before and after the final incubation of each lactobacilli strain but without mutans streptococci or candida. A pH-meter (Seven Easy pH, Mettler-Toledoo GmbH, Schwerzenbach, Switzerland) equipped with a flat electrode was used and the mean of two measurements was calculated.

### Statistical method

The data were processed with the SPSS software (version 17.0, Chicago Ill, USA) and subjected to chi-square tests. A p-value < 0.05 was considered as statistically significant.

## Results

### Characterization of lactobacilli and *Candida albicans*

Both *L. plantarum *strains as well as the *L. acidophilus *La5, *L. paracasei *F19 strains were identified by the API 50 CH system without difficulty. The two *L. reuteri *strains gave a biochemical profile interpreted as *L. fermentum*. None of the *L. rhamnosus *strains were fully identified by the system. All five candida strains were completely identified as *C. albicans *by the API Candida assay.

### Growth inhibition of mutans streptococci

The results of the growth inhibition assay are summarized in Table 2. At concentrations ranging from 10^9 ^to 10^5 ^CFU/ml, all lactobacilli strains inhibited the growth of the MS strains completely with the exception of *L. acidophilus *La5 that executed only a slight inhibition of some strains at concentrations corresponding to 10^7 ^and 10^5 ^CFU/ml. *L. acidophilus *La5 had a statistically significantly weaker inhibition capacity in comparison with the other probiotic strains (p < 0.05). At the lowest cell concentration (10^3 ^CFU/ml), only *L. plantarum *299v and *L. plantarum *931 displayed a total growth inhibition while a slight inhibition was seen for all five MS strains by *L. rhamnosus *LB21, *L. paracasei *F19, *L. reuteri *PTA 5289 and *L. reuteri *ATCC 55730. *L. rhamnosus *GG ATCC 53103 diluted to 10^3 ^CFU/ml inhibited the growth slightly for three of the five MS strains (*S. mutans *NCTC 10449, *S. sobrinus *OMZ176 and *S. mutans *P1:27) while low concentrations of *L. acidophilus *La5 did not affect MS growth. In general, no significant differences in growth inhibition were observed between the reference strains and the oral isolates.

**Table 2:** Growth inhibition of mutans streptococci strains by selected probiotic lactobacilli at different cell concentrations.

		Mutans streptococci (inhibition score)				
		
Lactobacilli	CFU/ml	Ingbritt	NCTC 10449	OMZ176	P1:27	P2:29
*L. plantarum *299v	10^3^-10^9^	0	0	0	0	0
*L. plantarum* *931*	10^3^-10^9^	0	0	0	0	0
*L. rhamnosus *GG	10^5^-10^9^	0	0	0	0	0
ATCC 53103	10^3^	0	1	1	1	0
*L. rhamnosus*	10^5^-10^9^	0	0	0	0	0
LB21	10^3^	1	1	1	1	1
*L. paracasei*	10^5^-10^9^	0	0	0	0	0
F19	10^3^	1	1	1	1	1
*L. reuteri*	10^5^-10^9^	0	0	0	0	0
PTA5289	10^3^	1	1	1	1	1
*L. reuteri*	10^5^-10^9^	0	0	0	0	0
ATCC 55730	10^3^	1	1	1	1	1
*L. acidophilus*	10^9^	0	0	0	0	0
La5 *	10^7^	0	1	1	0	1
	10^5^	1	1	1	1	1
	10^3^	2	2	2	2	2

Inhibition scores; 0 = total inhibition; 1 = slight inhibition; 2 = no inhibition.

* p < 0.05, chi-square test.

### Growth inhibition of *Candida albicans*

All the tested lactobacilli strains reduced candida growth but the effect was generally weaker than for mutans streptococci. Since an identical inhibition pattern was displayed for all candida strains, the results of the assays for only one of them are displayed in Table 3. At concentrations 10^9 ^and 10^7 ^CFU/ml, all lactobacilli except *L. acidophilus *La5 and *L. reuteri *PTA 5289 inhibited the five candida strains completely. At 10^5 ^CFU/ml, *L. rhamnosus *LB21, *L. rhamnosus *GG ATCC 53103, *L. paracasei *F19 and *L. reuteri *PTA 5289 displayed a slight inhibition, *L. acidophilus *La5 showed no inhibition at all while *L. plantarum *299v, *L. plantarum *931 and *L. reuteri *ATCC 55730 executed a total inhibition. At the lowest cell concentration, no inhibition was recorded except for the two *L. plantarum *strains.

**Table 3:** Growth inhibition of *Candida albicans *ATCC 28366 by selected probiotic lactobacilli at different cell concentrations.

Strain	Cell concentration (CFU/ml)			
	**10**^**9**^	**10**^**7**^	**10**^**5**^	**10**^**3**^
*L. plantarum *299v	0	0	0	1
*L. plantarum *931	0	0	0	1
*L. rhamnosus *GG ATCC 53103	0	0	1	2
*L. rhamnosus *LB21	0	0	1	2
*L. paracasei *F19	0	0	1	2
*L. reuteri *PTA 5289	1	1	1	2
*L. reuteri *ATCC 55730	0	0	0	2
*L. acidophilus *La5	1	1	2	2

Inhibition scores; 0 = total inhibition; 1 = slight inhibition; 2 = no inhibition.

### pH-measurements

The initial pH on the surface of the M17 and SAB agar plates were 6.8 and 5.8, respectively. After incubation, the pH decreased in all plates inoculated with lactobacilli and varied between 3.7 and 5.3 in the plates containing 10^3 ^CFU/ml (Table 4). *L. acidophilus *La5 displayed the highest pH values while the lowest values were recorded for the *L. plantarum *strains.

**Table 4:** Final pH on the surface of M17 and SAB agar plates after incubation with selected probiotic lactobacilli (10^3 ^CFU/ml).

Strain	M17 agar	SAB agar
*L. plantarum *299v	4.4	3.7
*L. plantarum *931	4.4	3.7
*L. rhamnosus *GG ATCC 53103	4.6	4.0
*L. rhamnosus *LB21	4.5	4.1
*L. paracasei *F19	4.8	4.4
*L. reuteri *PTA 5289	5.1	4.0
*L. reuteri *ATCC 55730	5.1	3.9
*L. acidophilus *La5	5.2	5.3

## Discussion

The predominant strategy for caries prevention relies on influencing the re- and demineralisation processes, mainly by using fluorides but also other factors involved in the caries process may be targeted [19]. One alternative could be to promote colonization of caries inhibiting bacteria and in this aspect, probiotic bacteria constitute a novel concept that needs further exploration. This *in vitro *study was performed to compare the effect of eight commercial probiotic lactobacilli strains on growth inhibition of mutans streptococci and *C. albicans*. We selected these particular lactobacilli strains because they are found in dairy products, fruit drinks, drops, gruels, chewing gums and tablets on the market. The test panel of mutans streptococci and yeast contained both reference strains and clinical isolates. *Candida albicans *is the most commonly isolated candida species in the oral cavity and constitute up to 80% of the clinical isolates [20]. Furthermore, candida has been ascribed a significant predictive value for dental caries in children [21]. The majority of the lactobacilli strains were fully identified with biochemical analysis using the API 50 CH system while a satisfactory identification of the *L*. *reuteri *and *L. rhamnosus *strains would require molecular genetic methods [22].

The agar overlay method was considered suitable for the research question since the method can test the inhibitory effect on multiple strains on a single plate. The method has earlier been used to demonstrate the *in vitro *growth inhibition of different isolates of lactobacilli on pathogenic bacteria in the female urogenital region [23] and to study the inhibitory effect of alpha-hemolytic streptococci on the otitis media pathogens *Streptococcus pneumoniae*, *Haemophilus influenzae *and *Moraxella catarrhalis *[24]. Kõll et al [25] used the deferred antagonism method to test the inhibitory capacity against mutans streptococci and *C. albicans*. In that method the lactobacilli are stab-inoculated on the surface of the bottom agar and pathogen suspensions are poured over the macrocolonies of lactobacilli. After incubation the width of the inhibition zones are measured. The main advantage with the deferred antagonism method is the more exact outcome while the agar overlay method enables assessment of different cell concentrations. Thus, the methods may be regarded as complements to each other.

With the exception of *L. acidophilus*, the present probiotic strains displayed strong inhibitory capacities against both the reference strains and the oral mutans streptococci isolates and these results were mainly in agreement with the recent observations of Simark-Mattsson and coworkers [17]. The findings concerning the candida strains were however novel and showed an even larger variation between the lactobacilli strains and only three of the eight tested strains exhibited strong inhibitory capacity. These results were in disagreement to Kõll et al [25] who failed to demonstrate inhibition of *Candida albicans *048 (wild-type). This could due to the method used in the experiments or the fact that the tested candida strain were more resistant then our reference strains and clinical isolates. The production of bacteriocins can be different in different systems and the ability to diffuse through the agar. Since significant growth inhibiting differences between the probiotic lactobacilli were noted, the null hypothesis was rejected. Similar strain-dependant differences have previously been observed concerning the metabolic capacity to form acids from dietary sugars that differs significantly between various probiotic strains [26,27].

The final pH in the medium has been suggested to be an important factor for growth inhibition, either directly or due to the production of bacteriocins at low pH [28]. We found a similar tendency in the present experiments; the lactobacilli strains that caused the lowest pH after incubation were also most effective in inhibiting MS and candida growth. The weakest inhibition of both mutans streptococci and *C.albicans *was displayed by *L acidophilus *La5 which also had the weakest acid production in both systems. In the agar overlay method the lactobacilli is grown inside the MRS-agar and it is possible that this particular system not is optimal for *L. acidophilus *La5. The inhibitory potential may be different, for better or for worse, in other test system or *in vivo*. However, also some bacteria with fairly weak acid production, such as *L. reuteri *ATCC 55730, proved to be effective against both mutans streptococci and candida. This indicates that other inhibitory substances also may be involved in the process with H_2_O_2 _being among the primary metabolites with inhibitory capacity against microbial pathogens [29]. The importance of H_2_O_2 _production for health can be illustrated in bacterial vaginosis, since lack of lactobacilli with such production seems to be associated with an altered composition of the vaginal flora resulting in a massive anaerobic overgrowth [30]. Tano et al. [31] concluded that the inhibitory effect of alpha-hemolytic streptococci on otitis media pathogens most likely was due to their H_2_O_2 _production. The antimicrobial glycerol derivative reuterin is another example of a growth inhibitory substance produced by *L. reuteri *[32] that may be involved in the reduction of mutans streptococci in saliva [14,15] as well as beneficial influences on gingivitis [33] and inflammatory mediators in gingival crevicular fluid [34]. Although any conclusion from *in vitro *studies must be drawn with caution, our findings provide some further support to the hypothesis that probiotic lactobacilli may influence the homeostasis and microbial profile in the oral cavity.

## Conclusion

The present *in vitro *study showed that commercial probiotic lactobacilli could inhibit growth of reference strains and oral isolates of mutans streptococci and candida but the capacity differed significantly between the strains. Further clinical studies are needed to verify whether or not the observed differences can play a role in the complex oral biofilm.

## Competing interests

The authors declare that they have no competing interests.

## Authors' contributions

PH contributed with the laboratory work, the acquisition, analysis and interpretation of data and took part in drafting of the manuscript. MH contributed with the design, laboratory work, analysis and interpretation of data and took part in drafting of the manuscript. ST contributed with drafting of the manuscript and revised it critically. CSB contributed with analysis and interpretation of data, drafting of the manuscript and revised it critically. All authors read and approved the final manuscript.

## Pre-publication history

The pre-publication history for this paper can be accessed here:

http://www.biomedcentral.com/1472-6831/10/18/prepub

## References

[B1] Meurman JH Stamatova I Probiotics: contributions to oral health Oral Dis 2007 13 443 451 10.1111/j.1601-0825.2007.01386.x 17714346

[B2] Twetman S Stecksén-Blicks C Probiotics and oral health effects in children Int J Paediatr Dent 2008 18 3 10 18086020 10.1111/j.1365-263X.2007.00885.x

[B3] Meurman JH Probiotics: do they have a role in oral medicine and dentistry? Eur J Oral Sci 2005 113 188 196 10.1111/j.1600-0722.2005.00191.x 15953242

[B4] Ouwehand AC Salminen S, von Wright A Antimicrobial components from lactic acid bacteria Lactic acid bacteria: microbiology and functional aspects 1998 2 New York: Marcel Dekker Inc 139 160

[B5] Allaker RP Douglas CW Novel anti-microbial therapies for dental plaque-related diseases Int J Antimicrob Agents 2009 33 8 13 10.1016/j.ijantimicag.2008.07.014 18804350

[B6] Becker MR Paster BJ Leyes EJ Moeschberger ML Kenoyon SG Galvin JL Boches SK Dewhirst FE Griffen AL Molecular analysis of bacterial species associated with childhood caries J Clin Microbiol 2002 40 1001 1009 10.1128/JCM.40.3.1001-1009.2002 11880430 PMC120252

[B7] de Carvalho FG Silva DS Hebling J Spolidorio LC Spolidorio DM Presence of mutans streptococci and Candida spp. in dental plaque/dentine of carious teeth and early childhood caries Arch Oral Biol 2006 51 1024 1028 10.1016/j.archoralbio.2006.06.001 16890907

[B8] Sissons CH Anderson SA Wong L Coleman MJ White DC Microbiota of plaque microcosm biofilms: Effect of three times daily sucrose pulses in different simulated oral environments Caries Res 2007 41 413 422 10.1159/000104801 17713343

[B9] Klinke T Kneist S de Soet JJ Kuhlisch E Mauersberger S Förster A Klimm W Acid production by oral strains of *Candida albicans *and Lactobacilli Caries Res 2009 43 83 91 10.1159/000204911 19246906

[B10] Badet C Thebaud NB Ecology of Lactobacilli in the Oral Cavity: A Review of Literature The Open Microbiology Journal 2008 2 38 48 10.2174/1874285800802010038 19088910 PMC2593047

[B11] Polonskaia MS Antibiotic substances in acidophil bacteria Mikrobiologiia 1952 21 303 310 14940720

[B12] Ahola AJ Yli Knuuttila H Suomalainen T Poussa T Ahlström A Meurman JH Korpela R Short term consumption of probiotic-containing cheese and its effect of dental caries risk factors Arch Oral Biol 2002 47 799 804 10.1016/S0003-9969(02)00112-7 12446187

[B13] Näse L Hatakka K Savilahti E Saxelin M Pönkä A Poussa T Korpela R Meurman JH Effect of long term consumption of a probiotic bacterium, Lactobacillus Rhamnosus GG, in milk on dental caries and caries risk in children Caries Res 2001 35 412 420 10.1159/000047484 11799281

[B14] Nikawa H Makihira S Fukushima H Nishimura H Ozaki Y Ishida K Darmawan S Hamada T Hara K Matwumoto A Aimi R Lactobacillus Reuteri in bovine milk fermented decreases the oral carriage of mutans streptococci Int J Food Microbiol 2004 95 219 223 10.1016/j.ijfoodmicro.2004.03.006 15282133

[B15] Caglar E Cildir SK Ergeneli S Sandalli N Twetman S Salivary mutans streptococci and lactobacilli levels after ingestion of the probiotic bacterium Lactobacillus reuteri ATCC 55730 by straws and tablets Acta Odontol Scand 2006 64 314 318 10.1080/00016350600801709 16945898

[B16] Caglar E Kuscu OO Selvi Kuvvetli S Kavaloglu Cildir S Sandalli N Twetman S Short-term effect of ice-cream containing Bifidobacterium lactis Bb-12 on the number of salivary mutans streptococci and lactobacilli Acta Odontol Scand 2008 66 154 158 10.1080/00016350802089467 18568474

[B17] Simark-Mattsson C Emilson CG Håkansson EG Jacobsson C Roos K Holm S Lactobacillus-mediated interference of mutans streptococci in caries-free vs. caries-active subjects Eur J Oral Sci 2007 115 308 314 10.1111/j.1600-0722.2007.00458.x 17697171

[B18] Hatakka K Ahola AJ Yli-Knuuttila H Richardson M Poussa T Meurman JH Korpela R Probiotics reduce the prevalence of oral candida in the elderly - a randomized controlled trial J Dent Res 2007 86 125 130 10.1177/154405910708600204 17251510

[B19] García-Godoy F Hicks MJ Maintaining the integrity of the enamel surface: the role of dental biofilm, saliva and preventive agents in enamel demineralization and remineralization J Am Dent Assoc 2008 139 Suppl 25S 34S 18460677 10.14219/jada.archive.2008.0352

[B20] Marsh PD Martin MV Oral Microbiology 2009 5 China: Elsevier Limited

[B21] Ollila PS Larmas MA Long-term predictive value of salivary microbial diagnostic tests in children Eur Arch Paediatr Dent 2008 9 25 30 18328235 10.1007/BF03321592

[B22] Nigatu A Evaluation of numerical analyses of RAPD and API 50 CH patterns to differentiate Lactobacillus plantarum, Lact. fermentum, Lact. rhamnosus, Lact. sake, Lact. parabuchneri, Lact. gallinarum, Lact. casei, Weissella minor and related taxa isolated from kocho and tef J Appl Microbiol 2000 89 969 978 10.1046/j.1365-2672.2000.01202.x 11123470

[B23] Rönnqvist D Forsgren-Brusk U Husmark U Grahn-Håkansson E Lactobacillus fermentum Ess-1 with unique growth inhibition of vulvo-vaginal candidiasis pathogens J Med Microbiol 2007 56 1500 1504 10.1099/jmm.0.47226-0 17965352

[B24] Tano K Grahn-Håkansson E Holm SE Hellström S Inhibition of OM pathogens by alpha-hemolytic streptococci from healthy children, children with SOM and children with rAOM Int J Pediatr Otorhinolaryngol 2000 22 185 190 10.1016/S0165-5876(00)00428-6 11137592

[B25] Kõll P Mändar R Marcotte H Leibur E Mikelsaar M Hammarström L Characterization of oral lactobacilli as potential probiotics for oral health Oral Microbiol Immunol 2008 23 139 147 10.1111/j.1399-302X.2007.00402.x 18279182

[B26] Haukioja A Söderling E Tenovuo J Acid production from sugars and sugar alcohols by probiotic lactobacilli and bifidobacteria in vitro Caries Res 2008 42 449 453 10.1159/000163020 18931494

[B27] Hedberg M Hasslöf P Sjöström I Twetman S Stecksén-Blicks C Sugar fermentation in probiotic bacteria - an in vitro study Oral Microbiol Immunol 2008 23 482 485 10.1111/j.1399-302X.2008.00457.x 18954354

[B28] Simark-Mattsson C Jonsson R Emilson CG Roos K Final pH affects the interference capacity of naturally occurring oral Lactobacillus strains against mutans streptococci Arch Oral Biol 2009 54 602 607 10.1016/j.archoralbio.2009.03.005 19394588

[B29] Dunne C O'Mahony L Murphy L Thornton G Morrissey D O'Halloran S Feeney M Flynn S Fitzgerald G Daly C Kiely B O'Sullivan GC Shanahan F Collins JK *In vitro *selection criteria for probiotic bacteria of human origin: correlation with *in vivo *findings Am J Clin Nutr 2001 73 386 392 10.1093/ajcn/73.2.386s 11157346

[B30] Verstraelen H Cutting edge: the vaginal microflora and bacterial vaginosis Verh K Acad Geneeskd Belg 2008 70 147 174 18669158

[B31] Tano K Grahn Håkansson E Wallbrandt P Rönnqvist D Holm SE Hellström S Is hydrogen peroxide responsible for the inhibitory activity of alpha-haemolytic streptococci sampled from the nasopharynx? Acta Otolaryngol 2003 123 724 729 10.1080/00016480310000403 12953772

[B32] Jones SE Versalovic J Probiotic Lactobacillus reuteri biofilms produce antimicrobial and anti-inflammatory factors BMC Microbiol 2009 11 9 35 10.1186/1471-2180-9-35 PMC2653509 19210794

[B33] Krasse P Carlsson B Dahl C Paulsson A Nilsson A Sinkiewicz G Decreased gum bleeding and reduced gingivitis by the probiotic *Lactobacillus reuteri* Swed Dent J 2006 30 55 60 16878680

[B34] Twetman S Derawi B Keller M Ekstrand K Yucel-Lindberg T Stecksen-Blicks C Short-term effect of chewing gums containing probiotic Lactobacillus reuteri on the levels of inflammatory mediators in gingival crevicular fluid Acta Odontol Scand 2009 67 19 24 10.1080/00016350802516170 18985460

